# Complicating cure: How Australian criminal law shapes imagined post‐hepatitis C futures

**DOI:** 10.1111/1467-9566.13562

**Published:** 2022-10-18

**Authors:** Kate Seear, Sean Mulcahy, Dion Kagan, Emily Lenton, Suzanne Fraser, Kylie Valentine, Adrian Farrugia

**Affiliations:** ^1^ Australian Research Centre in Sex, Health and Society La Trobe University Melbourne Victoria Australia; ^2^ Social Policy Research Centre University of New South Wales Sydney New South Wales Australia

**Keywords:** Alison Kafer, Australia, Bruno Latour, criminal law, hepatitis C, post‐cure

## Abstract

In recent years, highly tolerable and effective drugs have emerged promising a radical new ‘post‐hepatitis C’ world. Optimism about medical cure potentially overlooks discrimination and stigma associated with hepatitis C and injecting drug use. Legal frameworks are especially relevant to hepatitis futures, since the law has the potential to reinforce or alleviate stigma and discrimination. This article explores how hepatitis C figures in Australian criminal law and with what potential effects. Drawing on Bruno Latour’s work on legal veridiction, Alison Kafer’s work on futurity and disability and case law data collected for a major study on hepatitis C and post‐cure lives, we explore how the criminal law handles hepatitis C in the age of cure. We find that law complicates cure, constituting hepatitis C as disabling despite the advent of effective cures. The law steadfastly maintains its own approach to disease, disability and illness, untouched by medical and scientific developments, in ways that might complicate straightforwardly linear imaginaries of cure, transformation and progress of the kind that dominate biomedicine. We explore the implications of these tensions between law and medicine.

## INTRODUCTION

The blood‐borne virus hepatitis C is a major global public health challenge. Approximately 58 million people globally live with hepatitis C (World Health Organization, 2021), including 188,951 in Australia (MacLachlan et al., 2021). In Australia, transmission mainly occurs in the context of injecting drug use, via the sharing of needles and syringes or ancillary injecting equipment (Fraser & Seear, 2011). Over the last decade, drugs for the treatment of hepatitis C have emerged. Known as ‘direct‐acting antivirals’ (DAAs), these drugs have treatment regimens that are far shorter in duration (8–12 weeks) than previous drugs (24–48 weeks) and are more tolerable and effective, with rates of cure reported as being over 95% (European Association for the Study of the Liver, 2020). Such is the effectiveness of DAAs that the World Health Organization (WHO) has announced a goal to eliminate hepatitis C by 2030 (World Health Organization, 2016). In 2016, the Australian government announced that it would adopt the WHO’s elimination goal (Australian Department of Health, 2018) and made a substantial investment in DAAs, adding them to the nation’s subsidised prescription medication programme, the Pharmaceutical Benefits Scheme (PBS). This move promised access for all, with no restrictions according to disease stage, treatment history or drug use status.

Australia is considered an important case study for the viability of elimination (Scott et al., 2017). Direct‐acting antivirals have opened up the possibility of a radical new future without hepatitis C. They have also allowed us to imagine a future in which the lives of those once affected by the virus are radically transformed and greatly improved. For many people who have undergone treatment, this is indeed the case (e.g., Richmond et al., 2018). As we have argued elsewhere (Seear, Fraser, Farrugia et al., In press), however, we must take care not to overstate the effects of cure. Even if people do experience medical cure, for instance, an associated net of meanings attached to hepatitis C and injecting drug use can persist (Seear, Fraser, Farrugia et al., in press). Hepatitis C is a heavily stigmatised disease due, in particular, to its symbolic and practical association with injecting drug use (Fraser & Seear, 2011). Given this stigma, optimism about an imagined post‐hepatitis C future arguably ignores the many ways that hepatitis C continues to figure in law and policy, especially where laws and policies have not been updated to account for the advent of cure (Seear, Fraser, Farrugia et al., in press).

This article engages with these challenges. Our specific focus here is on how the advent of DAAs is being dealt with in one area of law in which hepatitis C features regularly: the Australian criminal law. In this article, we ask: How has the criminal law approached the virus in the age of highly effective and tolerable cures? Has the advent of DAAs changed the way the criminal law imagines the virus and its subjects? And what are the implications of the criminal law’s approach to the virus for an imagined, transformative, post‐hepatitis C future? We explore these questions by drawing on Bruno Latour’s (2013, 2009) work on legal veridiction and Alison Kafer’s (2013) work on disability as relational, alongside criminal law and crimes compensation law case data collected for an analysis of Australian case law that incorporates hepatitis C. We find that inherent in the criminal law is a focus on a future in which courts imagine how exposure to the virus might shape the prospects of both offenders and victims. By and large, we find, courts approach these futures with anxiety and trepidation, imagining them as potentially disabling despite the now ready availability of effective cure. Indeed, for cases decided after DAAs were added to the PBS, we find an almost total failure to consider what these treatments might mean and whether they are of any legal significance. These legal approaches undermine, we argue, the possibility of a radical new future in which hepatitis C is less stigmatised or stigmatising. We conclude with a discussion of the implications of these imagined and unimagined futures for people who have been cured of hepatitis C, looking closely at the notion of public imaginaries and the implications for other phenomena emerging in the context of hepatitis C (such as violence against women).

### Background and literature review

Hepatitis C was first named in 1989, having previously been described as non‐A, non‐B hepatitis (Fraser & Seear, 2011). The symptoms are uncertain and unpredictable but if chronic infection is left untreated, it can progress over time to cirrhosis of the liver or liver cancer (Fraser & Seear, 2011). For many years, the most widely available treatment for hepatitis C was known as ‘combination therapy’, made up of pegylated interferon and ribavirin. Combination therapy generated a wide range of serious side effects, including nausea, vomiting, hair loss, muscle pain, flu‐like symptoms, weight loss, anaemia, diarrhoea, anxiety, depression and reduced white blood count (Harris & Rhodes, 2013; Hopwood & Treloar, 2005). Many people discontinued treatment before the full treatment cycle was complete, and cure rates, which varied depending on genotype, were on average no more than 40% (European Association for the Study of the Liver, 2020). Both the disease and its treatment were disabling for many people. Indeed, in some parts of the world, including Australia, hepatitis C is a recognised disability, and people who have been diagnosed may become eligible for disability pensions or other entitlements, including protections under disability discrimination legislation (Seear, Fraser, Farrugia et al., in press; Seear, Fraser, Mulcahy et al., in press). Writing about combination therapy, Fraser and Seear (2011) argued that treatment pathways and experiences were also inseparable from the politics of injecting drug use and ‘addiction’ and that the treatment process was highly stigmatising. Such stigmatisation materialised in numerous ways including, notably, via dominant public health discourses, which drew on standard medical language to characterise those not cured as having ‘failed’ treatment. That is, responsibility for treatment ‘failure’ was attributed to the ostensibly ‘chaotic’ drug‐using lifestyles of those treated, rather than to medicine itself. Fraser and Seear (2011) suggested that any new treatments would lead to shifts in the relationships between medicine and its subjects, and to the lives of people diagnosed with hepatitis C, arguing for the importance of tracing such shifts as any new treatments emerged.

The advent of DAAs and the provision of universal access in Australia in 2016 created great excitement and expectation, generating hopes for a new, radically transformed future for people affected by the virus. When the drugs were listed on Australia’s PBS, the then federal Health Minister, Sussan Ley, described it as a ‘watershed moment’ in Australian health care (Gartrell, 2015). Since then, the rhetoric used to describe those treatments among experts and lay people is routinely positive. They have been described as a ‘gamechanger’ (Scott et al., 2017), and ‘one of the great advances in clinical medicine in recent decades’ (Dore, 2021, p. 36). They are said to have ‘revolutionised treatment’ (Banerjee & Reedy, 2016, p. 674), given their potential to ‘eliminate’ infection at the individual and population level and been described as a ‘therapeutic revolution’ (Rice & Saeed, 2014) and a medical triumph (Chung & Baumert, 2014; see also Dore & Martinello, 2020). This revolution metaphor has been questioned, however, in a recent analysis that finds that post‐cure lives are unlikely to be wholly transformed by medicine alone (Seear, Fraser, Farrugia et al., in press). The possibility that these treatments will usher in better futures for those cured is supported by research into improvements in psychological and physical wellbeing post‐cure (e.g., Goutzamanis et al., 2018; Richmond et al., 2018), but research also indicates that people have very high expectations of cure. They report hoping that it will allow them to make a symbolic break from their past (Richmond et al., 2018), feel more ‘normal’ and ‘worthy’ (Madden et al., 2018), enjoy a greater sense of social belonging (Harris, 2017) and experience less stigma. In other words, there is an expectation, at least among some, that medical treatments will generate powerful shifts in the social meanings attached to hepatitis C.

### Theoretical approach

These expectations bring to mind Alison Kafer’s ground‐breaking work on disability and cure, which we draw on in this article alongside Bruno Latour’s work on legal veridiction (2013). Kafer’s work (2013, p. 5) engages with key theories of disability, including both medical and social models, arguing that the medical model ‘frames atypical bodies and minds as deviant, pathological and defective, best understood and addressed in medical terms’. According to this model, disability is cast as ‘a problematic characteristic inherent in particular bodies and minds’ (2013, p. 5), rather than something shaped by social policies and processes. The social model critiques the medical model but ‘often relies on a distinction between impairment and disability’ that Kafer argues is problematic (2013, p. 7). Kafer thus prefers a ‘political/relational’ model of disability, which sees ‘the problem of disability [as] located in inaccessible buildings, discriminatory attitudes, and ideological systems that attribute normalcy and deviance to particular minds and bodies’. She argues that ‘The problem of disability is solved not through medical intervention or surgical normalisation but through social change and political transformation’ (Kafer, 2013, p. 6). Importantly, unlike the social model of disability (Shakespeare, 2006), this political/relational approach does not fix a distinction between impairment (as a physical or mental limitation) and disability (as the social meanings attributed to such impairment) but argues that ‘*both* impairment and disability are social’, such that ‘impairment doesn’t exist apart from social meanings and understandings’ (Kafer, 2013, p. 7; original emphasis). This approach differs from the social model in that it does not deny the benefits of cure, nor the ‘often‐disabling effects’ of bodies (2013, p. 7), but shares with the social model an emphasis on the constitution and maintenance of disability through social policies and processes. Key to our analysis here, Kafer’s formulation advances a co‐constitutive relationship between disability and the social.

Of course, the law is a social process and plays a vital role in constituting and maintaining disability. Kafer cites the *Americans with Disabilities Act (1990)* and the work of the United States Supreme Court as together constituting ‘disability’, thereby shaping both ‘normalcy’ and ‘deviance’. These approaches can, of course, change over time. It is surprising, then, given the central role of the law in the construction of disability, normalcy and deviance that academics have almost totally neglected the law’s relationship to hepatitis C and disability (although see Seear, Fraser, Mulcahy et al., in press). The work of Science and Technology Studies scholar Bruno Latour provides an especially useful framework for exploring these relationships, with its focus on how the law makes sense of the world, produces knowledge about it and shapes it. For Latour, the law is a ‘highly distinctive’ (2013, p. 54) structure that mobilises ‘its own [system of] explanation’ (2013, p. 359). In this sense:

law has its own separate place; it is recognized as a domain that can be isolated from the rest; it has its own force, as everyone would agree; and above all […] it has its own mode of veridiction, certainly different from that of science, but universally acknowledged as capable of distinguishing truth from falsity *in its own way*.

(Latour, 2013, pp. 358–359; original emphasis)

According to Latour, the law is the only mode of existence ‘to have offered […] resistance to the demand for explicitness’ (Latour, 2013, pp. 359–360), in that it is dominated by a simultaneously ‘obscure’ and ‘respectable’ method of knowledge and world‐making (see also Latour, 2009). In what follows, we bring Kafer’s interest in disability as relational together with Latour’s interest in law’s role in world‐making to explore how the law constitutes hepatitis C and its subjects. We consider how the law approaches hepatitis C, whether legal approaches have shifted with the advent of these treatments and what this means for the various expectations and hopes people have for medical cure, transformation and change.

## METHOD

For this project, we mapped legal, policy and practice frameworks as a means to identify all Australian statutes and case law that might impact on people with hepatitis C. Case law was gathered by a search of the open access Australasian Legal Information Institute Collection, using the term *hepatitis C*. We identified 1102 cases mentioning hepatitis C. Results were then screened for relevance and cases where hepatitis C had no bearing on the outcome or was mentioned only fleetingly were excluded. This left us with 232 cases with a substantive discussion of hepatitis C. Of these 232 cases, 98 involved crime, consisting mainly of either criminal cases where an offender was being sentenced (*n* = 80) or crime compensation cases (*n* = 18) where a victim was being compensated. Given the strong focus on hepatitis C in criminal cases, we chose to focus on these for the purpose of this article. Our other publications address hepatitis C in other areas of law (e.g., Seear, Fraser, Farrugia et al., in press; Seear, Fraser, Mulcahy et al., in press). The 98 criminal cases were decided between 1992 and 2020 were decided after the announcement that DAAs would be added to the PBS. In the analysis that follows, we explore how hepatitis C has historically featured in cases involving crime, and we attend to legal outcomes and the language used to describe hepatitis C and available treatments. In analysing these data, our specific interest is in how hepatitis C is imagined, constituted and veridicted under criminal law and what these legal approaches might mean for an imagined post‐hepatitis C world. We also attend to cases where legal veridictions evident before the advent of DAAs have been disrupted, contested or remade in the wake of these treatments.

## HEPATITIS C IN THE AUSTRALIAN CRIMINAL LAW

### Focus on futures

Hepatitis C appears in criminal cases for a wide range of reasons. This is largely an effect of the wide range of statutes and legal questions that are involved. When sentencing offenders, courts are generally concerned with punishment, rehabilitation and deterrence. When compensating victims, they are generally concerned with the recovery and rehabilitation of victims and with offering symbolic recognition of their pain and suffering. Despite these important differences, the questions that courts explore are interrelated. For instance, what should the court do when an offender assaults another person and either puts them at risk of acquiring hepatitis C or in fact transmits hepatitis C to them? Should actual transmission be treated as a more serious crime than possible (but ultimately unrealised) transmission? Is hepatitis C a ‘mild’, ‘moderate’ or ‘severe’ disease? And how might such categorisations inform the sentencing of the offender and any compensation payable to the victim? In some of the cases we analyse, it is the offender who has hepatitis C, while in others, it is the victim. Despite the many different scenarios, sero‐statuses (of offenders and victims), and legal outcomes in these cases, we find they are united by a *focus on futures*. By this we mean that specific legal minutiae, rules and practices oblige courts to consider how hepatitis C affects the prospects of both offenders and victims *in the future*. This includes a consideration of issues such as whether hepatitis C might reduce a person’s life expectancy, whether a person’s future will be significantly curtailed by the virus and what the effect of diagnosis might be on a victim’s psychological state.

In crime compensation cases, this focus on futures unfolds as a direct result of the need to assess, evaluate and ultimately quantify the impact of hepatitis C on the victim. There are two main possibilities for victims here. The first is that the victim acquires hepatitis C and the second is that they are only put at risk of acquiring it. In the crimes compensation cases we analysed (*n* = 18), no victims were diagnosed with hepatitis C after exposure. The focus was therefore exclusively on the period between exposure and confirmation that they did not have the virus. Here, victims frequently imagined their own futures, experiencing numerous fears and anxieties. It is clear from the case law that mental or nervous shock due to concern about the *possibility* of contracting the virus counts as an ‘injury’ for the purposes of victims of crime compensation (*Morris v Lowe* [2009] QSC 441). For instance, in *Northern Territory of Australia v Bentham* [1999] NTSC 119, a case that took place before the advent of DAAs, the offender bit a police officer. At a medical clinic, the police officer was advised that there was a risk he might acquire hepatitis C, although this advice did not appear to have been based on the offender actually having the virus. The victim suffered from anxiety for a period of about 10 months and eventually tested negative. The court suggested that the officer’s anxiety and psychological distress was ‘perfectly understandable’, with hepatitis C being described as ‘untreatable’ and ‘probably fatal’ and a ‘life threatening’ or ‘life‐terminating’ disease. There was evidence that the victim had ‘constant perseverating [repeating] thoughts that he may die’ and ongoing anxiety as a consequence. The victim was awarded compensation for ‘pain, suffering, and mental distress’. Another case shows that a victim need not spend as long as 10 months contemplating a possible future with hepatitis C to be entitled to compensation. Even a short period imagining that one might have the ‘dreaded disease’, as one judge called it (*Auton v Northern Territory of Australia* [2002] NSTSC 69), is enough. This was made clear in the case of *Calcutt v Letondeur* [2006] QDC 78, another case that took place before the advent of DAAs, which also involved an assault on police. Here, the offender was bleeding, and ‘lunged forward with his left arm and rubbed his blood up and down the [victim’s] right arm, indicating that he had AIDS and that he had Hepatitis C [*sic*]’. Importantly, the offender apologised to the victim the next day, told him he did not have any BBVs, and voluntarily took a blood test. That test confirmed that he did not have any BBVs. The court found that:

it would have been extremely distressing to the applicant to have another person’s blood rubbed on him and he must have been very upset for the short period of time during which he would have been uncertain about whether the respondent did, indeed, have a communicable disease such as AIDS or Hepatitis C.

The victim was awarded compensation for the trauma experienced: known in compensation law as ‘mental or nervous shock’. Crucially, courts generally do not assess whether a victim’s fears are logical or realistic. Even where the possibility of infection is deemed to be low, perhaps because the victim was spat on or bitten without the skin being broken, any anxieties experienced by the victim are treated as genuine and valid, routinely attracting compensation for mental or nervous shock (e.g., *Morris v Lowe* [2009] QSC 441; *Harman v Horne* [2001] QCA 349). In the only crimes compensation case decided since DAAs were added to the PBS, *James* [2018] WACIC 12, there was no mention of DAAs or their significance. The court accepted that potential exposure to the virus resulted in ‘psychological trauma’ and that the victim should be compensated.

In sentencing cases, the focus on futures is enabled by the minutiae of sentencing law, which obliges courts to consider various features of the offence and the offender. This includes identifying any ‘mitigating’ or ‘aggravating’ factors. Mitigating factors are those that are considered to merit a lesser penalty than the judge would have originally given but for the existence of those factors, whereas aggravating factors are those that are considered to merit a greater penalty than the judge would have originally given but for the existence of those factors. Relevant factors vary and can include features of the offending (e.g., that it was premeditated), personal circumstances of the offender (e.g., that they have dependant children) or personal circumstances of the victim (e.g., that they were vulnerable due to cognitive impairment or disability). In a small number of cases where the offender had hepatitis C, it had no effect on sentencing (e.g., *R v di Maria* [1996] SASC 6262; *R v Yu* [2003] NSWSC 1153). However, much more commonly, and over many years, courts have taken an offender’s hepatitis C status into account in sentencing (e.g., *Sant v R* [2014] NSWCCA 261; *R v Prideaux* [2009] VSCA 193; *R v CBK* [2002] NSWCCA 457). Courts differ, however, in the conclusions they draw about an offender having the virus. In some instances, the fact that an offender had hepatitis C mitigated their sentence (e.g., *R v Azar* [2000] NSWCCA 26; *R v Mueller* [1996] NSWSC 232). Historically, this was usually because the offender’s life expectancy was considered to be reduced by the disease or because it was disabling and would make their imprisonment ‘more burdensome than it would ordinarily be’ (*R v Johnstone* [2011]; see also *R v Glenbar* [2013] QCA 353; *DPP v Pittard* [2013] VCC 1150; *Sumner v R* [2010] VSCA 298; *Drury v The State of Western Australia* [2010] WASCA 220; *R v Orbach* [2007] VSCA 166). Importantly, this approach to sentencing persists now, even after the advent of DAAs (*Mitchell v R* [2018] VSCA 158; *DPP v Mitchell* [2017] VSC 423). In a number of other cases, the offender’s positive hepatitis C status was treated as an aggravating factor during sentencing, particularly where there was an assumed risk of transmission (e.g., *R v Leighton* [2014] QCA 169; *Police v Carter* [2002] SASC 48). For instance, in *R v BBK* [2008] QCA 2, which was a sexual offences case, the offender’s hepatitis C status was described as a ‘disturbing feature’ of his offending, although it did not appear that he had passed hepatitis C on to his victim, the disease is not generally classified as sexually transmissible, and there was no indication the offending was violent or drew blood. In *DPP (Acting) v Poole* [2015] TASCCA 10, the offender, who had hepatitis C, was arrested and taken to a local police station, at which point she became argumentative. Whilst in the police holding cell, she spat in a police officer’s face. While the risk of transmission of hepatitis C through saliva was described by the court as ‘minimal’, it was also held that the offence ‘would be viewed by the community with disgust and revulsion. It is made more serious when there is any consequent risk of infection’. Although the police officer did not acquire hepatitis C, and the virus is not medically considered transmissible by saliva (Bambridge & Stardust, 2018), the offender’s conduct was said to be aggravating. Other cases confirm that actual transmission is not required for it to be a ‘serious aggravating factor’ (*R v Robinson* [2007] QCA 349; see also *R v Sparks* [2010] NSWSC 1512). A similar conclusion was drawn in *Moore v Commissioner of Police* [2013] QDC 59, where an offender dug her fingernails into the wrist of an arresting police officer, after which she shouted that she had hepatitis C. The court believed those words, which ‘exacerbated’ the offence. Thus, suggesting that a victim *might* acquire hepatitis C is considered particularly egregious because it can generate anxiety and distress, causing them to contemplate a potential hepatitis C‐determined future. In a similar case, this forewarning of infection was said to have added ‘a rather nasty aspect to the assault’ (*Maslin v Police* [2006] SASC 333). The same result was found in *DPP v Gholikhani* [2016] VCC 2032, decided soon after the announcement that DAAs would be added to the PBS.

Hepatitis C can be an aggravating factor when the offending occasions an assumed risk of transmission to a victim, but it can be a mitigating factor when it impacts the health and wellbeing of the offender with hepatitis C in prison. Whilst seemingly inconsistent, we take the view that the law constitutes hepatitis C consistently, framing it as both *legally significant* and *inherently serious*. We say this for two reasons. First, references to the virus as ‘aggravating’ and ‘serious’ were made without further explication. We are routinely told that hepatitis C is grave. Second, victims were entitled to respond with ‘horror’ and ‘panic’ (*Ellery* [2006] WACIC 7) to possible exposure, regardless of how likely transmission was to occur. In both respects, hepatitis C futures are imagined and constituted as profoundly distressing and disabling. Significantly for our analysis, and as cases cited in the previous paragraph demonstrate, these descriptions were found both before and after the advent of treatments, with the advent of DAAs being of no legal or practical significance in terms of legal outcomes. Although medicine is routinely constituted as ‘revolutionary’, little attention is paid to the way that other disciplines or contexts might interfere with or complicate imagined revolutionary futures through maintenance of the status quo. In our analysis, hepatitis C bodies are, in Kafer’s (2013, p. 28) terms, figured as having ‘no future, as destined for decay, as always already disabled’ (Kafer, 2013, p. 34). Such imaginings are important, as Kafer (2013, p. 28) reminds us, because ‘the futures we imagine reveal the biases of the present’. As we have argued elsewhere, hepatitis C presents and futures are part of an ‘indivisible flow’ (Seear, Fraser, Farrugia et al., in press), constantly shaped by and shaping each other. In this sense, the criminal law’s biases constitute the virus in both the future and the present as something to fear, position hepatitis C futures as potentially catastrophic and constitute those who already live with the virus as living incomplete, perhaps even tragic lives. This is achieved by the law’s focus on futures and is sustained and stabilised through what we identify in the next section as *hierarchies* and *silences*.

### Hierarchies and silences

As we noted in the last section, hepatitis C is routinely and repeatedly constituted as a serious disease within criminal case law. Here, we argue that this designation is stabilised through *hierarchies* and *silences*. The plainest example of the former can be found in the case of *R v Heckendorf* [2017] QCA 59. This case was decided relatively early in the history of DAAs, but after the federal government announced that DAAs would be made publicly available for the treatment of hepatitis C. In this case, the victim met two men at a pub and later travelled to a house with them to continue socialising. When one of the men departed, the other, the offender, grabbed the victim, slamming her head against the floor. A struggle ensued. The offender then choked and raped the victim. In describing the victim’s injuries, the court said:

She suffered a fractured rib, injuries to her neck, arms and legs and bruising and a scratch to her head. *Most seriously, blood tests which were conducted over the next few days revealed that she had contracted Hepatitis C* [*sic* … ] In a statement tendered at the hearing, the complainant said that she had lost all self‐confidence [*sic*] and was unable to leave her house alone due to a ‘fear of people and large crowds’. She becomes agitated and nervous when left alone, which often causes her to break down and burst into tears. She feels very depressed and has had suicidal thoughts and feelings of worthlessness and shame. A letter from a doctor who has been treating her described the seriousness of Hepatitis C.

(Our emphasis)

Notably, the court produces a hierarchy of injuries and suffering, seemingly of its own account, in which the acquisition of the virus is said, without explanation, to be the assault’s ‘most serious’ consequence. This is despite the fact that the victim also suffered major injuries, which included psychological injuries that resulted in an inability to leave her own home. There is some possibility that the severity of her psychological injuries, in particular, were being downplayed. The evidence of a doctor on the apparent ‘seriousness’ of hepatitis C also appears to have been treated uncritically and there is no detailed discussion of whether this designation still holds in the DAA era. Notably, the hearing at which this letter would have been tendered occurred in March 2017, after the addition of DAAs to the PBS. The only reference to cure positions it as relevant to her future, but without imagining it in the way medicine does (i.e., as profoundly transformative):

The condition can be treated and cured, but the medication is expensive and would require her to travel a considerable distance to the nearest Hepatitis C clinic, which is in [a regional centre]. There are side effects from the treatment, most commonly flu‐like symptoms, low blood counts, difficulty in thinking, nervousness and depression.

Here, the court emphasises structural factors such as limited access, relative cost and inconvenience to underscore the challenges of treatment—factors that may be lessened since the advent of less invasive treatment. To be clear, we do not discount the seriousness of the injuries sustained by the victim nor do we question the profound distress she experienced as a result of the assault or that acquiring hepatitis C in this way would likely be traumatic. Our point is that the court’s description of treatment and the hierarchy it appears to impose on the victim’s injuries renders her suffering untouched by the advent of cure. Put simply, the law retains its longstanding view of hepatitis C as an extremely serious disease. This designation has several potential effects. It may, as in this case, lead to a greater sentence for the offender. Importantly, it may also compound the victim’s suffering, contributing to a sense that she faces a debilitating or bleak future. It also helps to sustain the stigma associated with hepatitis C even in the elimination era.

There are other ways that these constructions of the virus can operate symbolically and materially to the detriment of victims. The clearest example of this is the case of *DPP v Ljepojevic* [2013] VCC 238. Here, the offender had threatened to kill his pregnant former partner after they had separated. Shortly after issuing the threat, he attended her house and viciously assaulted her, including by strangulation. Earlier, the victim had alleged on social media that she would prevent the offender from seeing their children and that he had hepatitis C. In sentencing the offender, the court agreed that the victim’s online allegations could be considered as a ‘provocation’. The notion that victims, particularly women, might provoke their attackers, and that this should reduce an offender’s culpability, is highly controversial (Morgan, 1997). Feminists have long argued that provocation‐type arguments excuse men’s violence (Morgan, 1997). Here, we see a different kind of sequencing effect, in the sense that the provocation finding reorders agency and responsibility, reducing his culpability and assigning some blame to the victim. Although it is not said explicitly, we argue that the court takes this view because hepatitis C is a highly stigmatised virus, and therefore, a likely source of horror and shame. Indeed, in sentencing the offender, the presiding judge stated that ‘I accept that to some degree you were provoked’; while acknowledging that the assault involved ‘blatant violence’, the judge also said that the offender ‘had little control over [his] actions’. The court did not address whether the offender had hepatitis C; instead, the allegation that he had the disease (or perhaps, the public disclosure of his status) is treated as triggering a loss of control. While a coronial inquest into his subsequent death in prison found that he had a history of hepatitis C (*Inquest into the Death of Jason Joseph Mirko Ljepojevic* [2018] VicCorC 24510), this is not addressed in his sentencing. Although public understandings of male violence may have shifted in the years since *DPP v Ljepojevic* was decided, no criminal law cases have reopened, revisited or revised the connections made in that case. As such, we conclude that hepatitis C figures as a source of symbolic horror and shame (see Fraser & Seear, 2011) and that courts are at times disposed towards being merciful to those publicly accused of having it even when their violent reactions to such accusations are wildly disproportionate.

These constructions of hepatitis C as serious and stigmatising are underscored by another feature of the case law: *silences* regarding the advent of these treatments. As we noted earlier in this article, the 98 cases we collected and analysed here unfold across a wide time frame, both before and after the advent of DAAs. Given the implicit (and sometimes explicit) focus on futures, including medical prospects and anxieties, we might expect to have seen a deliberate and concerted focus, in the post‐DAA cases, on these treatments and their potential significance for the prospects of both victims and offenders. We find virtually no discussion of them in the case law, however, and legal depictions of the virus that pre‐date DAAs are not reopened, revisited or revised in the wake of these treatments (e.g., *DPP v Tran* [2018] VCC 1222; *DPP v Eastwood (a pseudonym)* [2017] VCC 1739). Only one case, which we discuss in more detail in the next section, offers a comprehensive assessment of their potential legal significance. These silences are made more significant by the fact that a small number of historic cases did discuss the old treatments in detail. In *R v Peters* [2002] NSWSC 1234, for instance, the offender had both hepatitis C and HIV. There was a lengthy discussion in this case of the old treatments, treatment options and complications for both viruses, how the two viruses might interact and the relevance of this to treatment options and success. In another case, *Orchard v R* [2004] WASCA 23, the offender had committed armed robbery and theft of a motor vehicle. Sometime before this offending, he had been treated with the old (interferon‐based) treatments. He argued that these had caused numerous side effects and that this reduced his moral culpability, leading to a personality change, which affected his reasoning processes and decision‐making, contributing to the commission of the offence. There was evidence from the offender’s ‘hepatitis co‐ordinator’ at a metropolitan hospital that the medication had a ‘psychotic component’. Accepting this, the court reduced the offender’s sentence from that which was initially deemed appropriate, if interferon had not been a factor. In total, his prison time was reduced by 4 years. A series of more recent cases demonstrate the ability of courts to be responsive and agile in the face of ‘rapidly evolving’ (*Brown v R* [2020] VSCA 60) medical developments. After the advent of the COVID‐19 virus and resultant pandemic in early 2020, courts across the country have been asked to take it into account when sentencing offenders to avoid the exposure of offenders to the virus in prison settings. Related to this, some courts have been asked to reduce sentences on the basis that sentencing a person with hepatitis C to prison would increase their risk of acquiring COVID‐19 or increase the risk of serious illness as a result of the combination of the viruses (e.g., *R v Debortoli* [2020] NSWDC 466; *R v Nolan* [2020] VSC 416; *R v Awad* [2020] NSWDC 711).

Two observations can be made here. First, courts have previously shown a willingness and ability to grapple, sometimes in great detail, with the nature and effects of hepatitis C treatments. This includes at least one case, *Orchard*, where the court wrestled with complex and weighty questions regarding the putative effects of treatment on thought processes and conduct, with implications for how we understand agency and responsibility. Second, while courts have a proven ability to respond swiftly to medical and scientific developments, as with the example of COVID‐19, this agility is not evident in the case of hepatitis C and DAAs. Why have courts not engaged meaningfully with these treatments? We can only speculate here, but it could be the result of lawyers and/or judges not being aware of developments pertaining to treatment. The key point is that the law continues to imagine and indeed *do* hepatitis C ‘*in its own way*’ (Latour, 2013, pp. 358–359; original emphasis), in the sense that it responds swiftly to one virus but not to developments regarding another. Whatever the reason for them, these silences have the effect of preserving hepatitis C as a serious and disabling condition and a legitimate source of anxiety and concern, untouched by medical and scientific developments. On the few occasions where these treatments were explicitly mentioned in the case law, the seriousness of hepatitis C was simply reiterated. As we flagged earlier, only one case offered a comprehensive and rigorous assessment of what these treatments might mean. We consider this case in detail in the next section.

### Grappling with the advent of cure

The only case we identified that grapples in depth with the advent of these treatments is the widely publicised criminal case known as *Peters v R* (No 2) [2019] VSCA 292. The case, which occasioned significant media interest, concerned an anaesthetist, James Peters, who had hepatitis C. Between 2006 and 2009, Peters worked as an anaesthetist at the Croydon Day Surgery, which is a service that provides pregnancy terminations in the eastern suburbs of Melbourne, Victoria. From June 2008 to November 2009, Peters stole syringes of fentanyl, injected himself with it and then re‐used the syringes on patients. At least 55 of his patients were infected with hepatitis C, with these infections attributed to Peters.^1^ Of these, about 8–10 tested positive for hepatitis C antibodies but negative for the virus itself, meaning that by the time of testing they had ‘cleared’ the virus, meaning their bodies had eliminated the virus without treatment. Some women did not know they had been infected. Some patients were also asymptomatic. In March 2013, Peters pleaded guilty to 55 charges of ‘negligently causing serious injury’ and was sentenced to 14 years’ imprisonment. This offence is found in section 24 of the *Crimes Act 1958* (Vic). The wording of the provision has since changed, but at the time it stated that:

A person who by negligently doing or omitting to do an act causes serious injury to another person is guilty of an indictable offence.

(Crimes Act 1958 (Vic))

Injury was defined as that which ‘includes unconsciousness, hysteria, pain and any substantial impairment of bodily function’, and serious injury was defined as that which ‘includes a combination of injuries’. In late 2015 or early 2016, Peters learnt, whilst in prison, about these (then new) hepatitis C treatments and himself underwent treatment. This led him to consider whether the advent of these more tolerable and effective treatments were of legal significance. Had he really caused those women a ‘serious injury’ if hepatitis C was now readily curable? What if those women had in fact now been cured? Might that change his crime? In June 2018, Peters asked the court to consider these questions, lodging an appeal against his original sentence. His argument, in short, was that because these treatments had a success rate of close to 100%, persons who take these treatments will not develop ‘long term injurious symptoms of hepatic infection’. In this sense, hepatitis C was no longer constituted as a ‘serious injury’; and if these treatments had been available at the time of trial, he argued that he would not have pleaded guilty to the charge. The case was therefore quite centrally concerned with the advent of DAAs and whether this fundamentally changes how the law should view hepatitis C.

At the original trial, the sentencing judge said that Peters gave victims ‘a virus from which there is no certain recovery’ and which caused ‘significant emotional trauma’ (*R v Peters [2013] VSC 93*). At the appeal, Peters’ lawyers argued that it was only when the injurious consequences of infection manifest that the virus causes injury and that actual damage needed to be shown. In response, the prosecution argued that at the time of conviction, serious injury had a broad meaning. The prosecution also argued that it was ‘reasonably open to regard the fact of being infected with a virus such as hepatitis C, that permeates every bodily cell of the victim, as a serious injury’. This was so even for those who had cleared the virus. On this, the prosecution argued that:

the question of seriousness fell to be decided as at the time when the injury was suffered, namely the time of infection. At that point, it was not known whether the person who suffered the injury would experience symptoms at a later date or ‘clear’ the virus, but the potential impact of the infection meant that it could readily be regarded as ‘serious’.

Various symptoms and side effects of treatment were then described, with the court noting that:

These impacts were to be anticipated even without considering the potentially life‐threatening consequences of the virus, including cirrhosis, liver failure and liver cancer.

Quoting from an important precedent, the court noted that ‘injury’ and ‘serious’ are ordinary words to be considered by a jury. Importantly, ‘seriousness’ involved a value judgement and was a comparative exercise. Serious injuries are identified by comparison to injuries which, ‘according to ordinary human experience, [would] be commonly regarded as slight, superficial or trifling’ (per Brooking J in the earlier case of *R v Rhodes* (1984) A Crim R 124). The court also noted that it was clear that physical injury was not needed, because of the presence of the word ‘hysteria’ in the definition of injury in the Act. On seriousness, the court said that the ‘degree of likelihood of future consequences, and the nature of those potential consequences […] properly bear on the question whether the injury is “serious”’.

The court went on to say that ‘seriousness is to be determined at the time of the injury [basically at the time of the infection], albeit informed by hindsight given current events. The question is whether the injury itself is “serious”, not whether it has had serious consequences’. To illustrate this point, the court noted that if a person breaks their leg, it matters not that the leg can heal. The availability of treatment cannot ‘undo’ the seriousness of the injury. As such, the court rejected Peters’ appeal. They concluded that:

the evidence does nothing to detract from the seriousness of the injuries at the time they were caused […] that conclusion was open irrespective of the prospects of remedial medical treatment on the evidence as it stood at the time. The analysis is unchanged by the availability (or receipt) since then of more effective treatment. *Even if it could be shown that the adverse effects of hepatitis C can be wholly avoided without significant side effects, that would not alter the fact that those adverse effects were the prospective consequences of the infection, and the gravity of their nature would have sufficed to justify finding the injuries to be serious.*

(Our emphasis)

The acquisition of hepatitis C was similarly held to be a ‘serious injury’ in another Victorian case (*DPP v McMillan* [2019] VCC 1611). In this case, which was decided 3 months before the second *Peters* appeal, a general practitioner refused to provide an opioid painkiller prescription to the offender who had hepatitis C and a lengthy history of illicit drug use. The offender then assaulted the general practitioner, spitting on and hitting him, and this was attributed as the cause of the doctor acquiring hepatitis C. As a result of the infection, the victim ‘was not able to practice as a doctor for some time because of the possibility of infecting others and he had to undergo prolonged treatment’ [para 46]. The sentencing judge stated that ‘I am not aware of any similar case and counsel was not able to direct me to any case where the serious injury caused was hepatitis C’. Oddly, the original *Peters* sentence and appeal were not cited, and there is no discussion of the advent of these treatments for hepatitis C. It was noted by the sentencing judge that ‘it is fortunate for him that medical testing shows that he is no longer infected with the hepatitis C virus’.

The Peters case and other case law confirm that the putative biomedical significance of these treatments is of little consequence under criminal law. The criminal law affords judges considerable flexibility, in moving beyond temporal logics, symptoms, cures and other material factors to continue to constitute the virus as inherently grave. Hepatitis C is enacted as a particular kind of problem, without distinction between those who were asymptomatic, had cleared the virus, had not known they had it and experienced severe symptoms. Importantly, as we have argued elsewhere, the *Peters* case demonstrates the law’s ‘potential to generate new harms by stigmatising all those with hepatitis C, those cured, and those at risk, even as [it] recognises the women infected with it as victims of a terrible wrong’ (Seear, Fraser, Mulcahy et al., in press). It is important to acknowledge the constitutive work being done by the uncertainty of disease progression here, as well as anticipation or supposition: The *mere anticipation* of symptoms or side effects can be enough to render something serious. The result we get in the *Peters* case is arguably underpinned by sound public policy rationales. For instance, the notion that we should treat injuries as serious without regard for how those injuries might, in theory, be treated, ensures that offenders are punished for the *possible* consequences of their actions, regardless of any steps that victims subsequently take (or not) to limit the extent of those injuries. Nevertheless, in Latour’s terms, we think of this as a kind of steadfastly distinct approach to disease veridiction; one which is unmoored from medical and scientific developments, unconcerned with them and capable of producing, reproducing or disrupting how health and illness is dealt with in other contexts, by other disciplines or institutions.

## CONCLUSION

This article has explored how hepatitis C features in the Australian criminal case law, including before and after the advent of DAAs for the treatment of hepatitis C. We also considered whether the arrival of much more tolerable and effective treatments have resulted in a legal re‐imagining of the virus and its subjects and considered some of the effects of these legal imaginings. Overall, we found that the law maintained a consistent approach to hepatitis C. Essentially undeterred by the advent of these treatments, the law has made no significant revision to the way it conceptualises the virus. Instead, the criminal law continues to allow for and indeed concretise a view of both potential exposure to and acquisition of hepatitis C as inherently ‘serious’. Hepatitis C is enacted as a particular kind of problem, without distinction between those who are asymptomatic, had cleared the virus and those who experienced severe symptoms. Put another way, we find that the distinction between infection (sometimes referred to as the point of actual or perceived ‘injury’) and disease (having chronic infection that might lead to serious illness if left untreated) is consistently blurred in law or rendered irrelevant for the purpose of legal assessments, sentencing and compensation. These constructions matter for several reasons. They have the power to shape public understandings of hepatitis C *infection as disease*, constituting the virus as inevitably dangerous and serious, despite the advent of cure. These legal imaginings also have the potential to shape how people with the virus understand themselves, as well as their ability to avoid the stigma associated with it. People who have hepatitis C, or have been exposed to the virus, may be further ‘enroled’ into a discourse (following Butler, 1997, after Althusser, 1971) where hepatitis C is serious, significant and shameful.

Of course, it is important to make the point that legal discourses do not inaugurate stigma and are not responsible for stigma on their own. Hepatitis C continues to be stigmatised outside law. For instance, in other work, we have found that the effects of judgement and stigma can persist even post‐cure, with the lingering presence of antibodies sometimes interpreted as proof the person still ‘has’ the virus and is ‘infectious’, thus posing a ‘risk’ to others (Seear, Fraser, Mulcahy et al., in press). We have also argued that biomedical imaginaries can help to generate and sustain stigma (Seear, Fraser, Mulcahy et al., in press). That is, various forces and factors, including poverty and homelessness, shape access to treatment (Farrugia et al., 2022). Excessive optimism regarding the ease and accessibility of cure risks generating stigma because it might enable criticism of those who have not undergone treatment. Our point here is that stigma is not solely the product of law, but is sustained by a complex range of forces. Nevertheless, the law can reproduce, magnify and exacerbate the stigmatisation of hepatitis C. The law steadfastly maintains its own approach to infection, injury, disease, disability and illness. It produces a ‘highly distinctive’ (Latour, 2013, p. 54) approach to hepatitis C, by materialising ‘its own [system of] explanation’ (Latour, 2013, p. 359), its own mode of veridiction. On occasion this includes conflating infection with disease and with reduced quality of life. Persistent ideas about the virus and those who have it become difficult to dislodge in the face of the criminal law. To return once more to Kafer (2013, p. 2), we argue that legal ‘assumptions about the experience of disability create one’s conception of a better future’. People may come to understand their presents and futures as substantially constrained by hepatitis C infection or as a source of deep anxiety and trepidation. These ways of imagining the virus and its futures fold back into law (for example, in crime compensation cases). In other words, people evince a certain ‘attitude’ (Kafer, 2013, p. 6) towards infection with the virus as inevitably disabling, which is concretised through law, and repeated back to hepatitis C’s subjects. The disabling effects of hepatitis C are co‐constituted, following Kafer, through law’s circular, sealed processes. Our concern is with a system that perpetuates a particular understanding of hepatitis C and with that system’s potential to generate and exacerbate stigma and distress as a consequence of these approaches.

These effects are made possible by the law’s specificities, concerned as it is with compensating victims and punishing and rehabilitating offenders. These legal minutiae are not easily reformed and, in many instances, are underpinned by sound public policy rationales. Nevertheless, our key point is that the criminal law complicates or interferes with straightforwardly linear imaginaries of cure, transformation and progress of the kind that dominate in biomedicine. The criminal law reinscribes assumptions about the permanency and perniciousness of the virus, failing to accommodate or account for recent medical developments in treatment and cure. We need to address all the forces that can shape people’s lives, and in turn, impact on the elimination agenda (Seear, Fraser, Farrugia, et al., in press). We need a much more explicit emphasis on legal and policy environments that sustain and perpetuate stigma, as well as those—such as the media—responsible for reporting on legal outcomes and shaping public understandings of them.

## AUTHOR CONTRIBUTIONS

**Kate Seear**: Conceptualisation (Lead); Formal analysis (Lead); Funding acquisition (Equal); Methodology (Equal); Supervision (Lead); Writing—original draft (Lead). **Sean Mulcahy**: Data curation (Equal); Formal analysis (Supporting); Investigation (Lead); Writing—review & editing (Equal). **Dion Kagan**: Formal analysis (Supporting); Project administration (Supporting); Writing—review & editing (Supporting). **Emily Lenton**: Formal analysis (Supporting); Project administration (Supporting); Writing—review & editing (Supporting). **Suzanne Fraser**: Formal analysis; Supporting, Funding acquisition (Equal); Methodology (Equal); Writing—review & editing (Equal); Kylie Valentine: Formal analysis (Supporting); Funding acquisition (Equal); Methodology (Equal); Writing—review & editing (Supporting). **Adrian Farrugia**: Formal analysis (Supporting); Funding acquisition (Equal); Methodology (Equal); Writing—review & editing (Supporting).

## Data Availability

All data are derived from public domain resources at austlii.edu.au. The data are not supported by a reference number and the data that support the findings of this study are available from the corresponding author upon reasonable request.

## ENDNOTE

^1^
We say ‘at least’ here because although the criminal case refers to 55 affected women, separate civil class action refers to a larger class of victims (60–62). See *A v Schulberg* (No 2) [2014] VSC 258.
